# Transcriptomic analysis of rat prefrontal cortex following chronic stress induced by social isolation – Relevance to psychiatric and neurodevelopmental illness, and implications for treatment

**DOI:** 10.1016/j.ynstr.2024.100679

**Published:** 2024-10-17

**Authors:** Jen-Yin Goh, Patricia Rueda, Joy Taylor, Alex Rathbone, Daniel Scott, Christopher J. Langmead, Kevin C.F. Fone, Gregory D. Stewart, Madeleine V. King

**Affiliations:** a Neuromedicines Discovery Centre, Monash Institute of Pharmaceutical Sciences, Monash University, Parkville, Victoria, Australia; b School of Life Sciences, University of Nottingham, Medical School, Queen's Medical Centre, Nottingham, NG7 2UH, UK

**Keywords:** Stress, Social isolation, Rat, Gene expression, Prefrontal cortex, Muscarinic M_4_ receptor, Nuclear receptor 4A1

## Abstract

Social isolation is an established risk factor for psychiatric illness, and became increasingly topical with the spread of SARS-CoV-2. We used RNA sequencing (RNA-Seq) to enable unbiased assessment of transcriptomic changes within the prefrontal cortex (PFC) of isolation-reared rats. To provide insight into the relevance of this manipulation for studying human illness, we compared differentially expressed genes (DEGs) and enriched biological functions against datasets involving post-mortem frontal cortical tissue from patients with psychiatric and neurodevelopmental illnesses. Sixteen male Sprague-Dawley rats were reared in groups of four or individually from weaning on postnatal day (PND) 22–24 until PFC tissue collection for RNA-Seq (PND64-66). We identified a total of 183 DEGs in isolates, of which 128 mirrored those in PFC tissue from patients with stress-related mental illnesses and/or neurodevelopmental conditions featuring social deficits. Seventy-one encode proteins classed as druggable by the gene-drug interaction database. Interestingly there are antagonists or inhibitors for the products of three of these up-regulated DEGs (*Hrh3*, *Snca* and *Sod1*) and agonists or activators for products of six of these down-regulated DEGs (*Chrm4*, *Klf2*, *Lrrk2*, *Nr4a1*, *Nr4a3* and *Prkca*). Some have already undergone pre-clinical and clinical evaluation, and studies with the remainder may be warranted. Changes to *Hrh3*, *Sod1*, *Chrm4*, *Lrrk2*, *Nr4a1* and *Prkca* were replicated in an independent cohort of sixteen male Sprague-Dawley rats via quantitative reverse transcription polymerase chain reaction (qRT-PCR). Our findings support the continued use of post-weaning isolation rearing to investigate the neurobiology of stress-related disorders and evaluate therapeutic targets.

## Highlights

• Social isolation is a topical risk for mental illness and can be replicated in rats.
• Resulting PFC transcriptomic changes overlap those in psychiatric disease.
• There are modulators for several of our up- and down-regulated genes.
• Pre-clinical testing of these modulators in stress-induced models appears warranted.

## 1 Introduction

Social adversity, deprivation and stress are linked to schizophrenia, bipolar disorder (BD), major depressive disorder (MDD), anxiety and substance abuse. For example, parental separation or loss, abuse or bullying during childhood, frequent relocation during adolescence and social disadvantage or exclusion extending into later life are all established risk factors for individual disorders or their comorbidity (Cerdá et al., 2010; Stilo and Murray, 2010). These issues have become increasingly relevant with the recent spread of SARS-CoV-2. Chronic psychosocial stress can be replicated in rodents by post-weaning isolation of gregarious rats in individual cages lacking environmental enrichment. The resulting absence of physical contact and play throughout adolescent neurodevelopment causes a range of long-term behavioural, structural, neurochemical and molecular changes in experimental animals that emerge post-puberty and have apparent translational relevance to psychiatric disorders like schizophrenia, which this particular stressor has traditionally been claimed to model (for reviews see Fone and Porkess, 2008; Jones et al., 2011). In summary, isolates exhibit hyper-reactivity to novel environments and drugs of abuse (including amphetamine and cocaine), altered social interaction and increased aggression, plus an array of cognitive deficits across multiple domains (pre-attentional processing, visual and associative learning and memory, reasoning and problem solving). These are accompanied by mesolimbic dopamine hyperfunction, mesocortical dopamine hypofunction and changes to a variety of GABAergic and glutamatergic markers (Heidbreder et al., 2000; Jones et al., 2011; Shortall et al., 2020). We and others have also identified changes in the gut microbiome (Dunphy-Doherty et al., 2018) as well as brain regional cytokines (Shortall et al., 2018; Goh et al., 2020), metabolism (Sun et al., 2021) and responses to inflammatory immune challenge (Russell et al., 2022) that are all topical areas for mental health research. Some aspects of the isolation syndrome are relevant to the positive symptoms of schizophrenia (which generally respond well to available antipsychotics) while others can offer valuable insight into the neurobiology of negative and cognitive symptoms plus associated comorbidities, and enable preclinical evaluation of novel therapeutics for these poorly-managed features of the disorders we seek to model.

It is widely acknowledged that perturbed function of the prefrontal cortex (PFC) contributes to the development of psychiatric and neurological disorders (Goto et al., 2010), and the PFC is clearly a key locus of isolation-induced change in experimental models. Cognitive deficits in isolated rats encompass fear conditioning (e.g. Weiss et al., 2004; Mora-Gallegos and Fornaguera, 2019), reversal learning (e.g. Jones et al., 1991; Schrijver et al., 2004; Powell et al., 2015) and attentional set shifting (Schrijver and Würbel, 2001; McLean et al., 2010), which are regulated by the PFC (Birrell and Brown, 2000; McAlonan and Brown, 2003; Rozeske et al., 2014). In addition, isolates exhibit a selective 5–7% decrease in PFC volume (Day-Wilson et al., 2006; Schubert et al., 2009), diminished serotonergic function (Bickerdike et al., 1993) and altered GABAergic responses to pharmacological challenge (Napolitano et al., 2014). At the molecular level a variety of research groups describe isolation-induced changes in medial PFC expression of immediate early genes and those involved in apoptosis, cell differentiation and glutamatergic signalling (Levine et al., 2007; Hermes et al., 2011), including elevated expression of mRNA encoding the NMDA receptor NR2A subunit (Turnock-Jones et al., 2009) and down-regulation of that encoding the NR2B subunit (Loureiro et al., 2020) and neuregulin-1 receptor ErbB4 (Gilabert-Juan et al., 2013). Additional research has focussed on genes encoding dopamine and 5-HT receptors (Ko and Liu, 2016), calbindin (a calcium binding protein; Gilabert-Juan et al., 2013), brain-derived neurotrophic factor (Li et al., 2016), prolyl endopeptidase (a serine protease; Zubkov et al., 2019), NKCC1 and KCC2 (ion co-transporters that regulate GABAergic function; Atefimanash et al., 2021), purinergic receptors (Andrejew et al., 2021), cytokines (Ko and Liu, 2016; Corsi-Zuelli et al., 2019) and inflammatory markers, some of which are normalised by chronic administration of atypical antipsychotics (Amiri et al., 2021).

However, the aforementioned studies either only looked at a selected number of genes directly relevant to a specific hypothesis, or were restricted by technical constraints of the approach, for example relatively low resolution, high background noise and limited range of discoverable genes in the case of DNA microarrays (Li and Teng, 2016). RNA sequencing (RNA-Seq) utilises high-throughput next-generation sequencing to enable unbiased assessment of expression changes across the entire transcriptome and to detect alternatively spliced genes, rare transcripts and non-coding RNAs in disease or models thereof (Li and Teng, 2016). It has comparable sensitivity to quantitative reverse transcription polymerase chain reaction (qRT-PCR) techniques and there is generally a good correlation between the two approaches (Everaert et al., 2017). RNA-Seq has provided recent insights into lasting transcriptomic consequences of short-term (two weeks) social isolation followed by a longer period of re-socialisation (three weeks), which point towards changes in oxidative phosphorylation (Sun et al., 2018, 2021). But because re-socialisation can overcome components of the isolation syndrome (Gentsch et al., 1988; Tulogdi et al., 2014), this study used RNA-Seq to identify additional genes that are differentially expressed at the time of ongoing and more prolonged psychosocial stress. To provide insight into the relevance of this manipulation for studying human illness we compared differentially expressed genes (DEGs) and enriched biological functions against those in published studies or open access datasets involving post-mortem frontal cortical tissue from patients with schizophrenia, BD, MDD and autistic spectrum disorder (ASD), and identified shared DEGs that might represent therapeutic targets. We confirmed expected development of the typical isolation syndrome prior to RNA-Seq by monitoring body weight (which increases upon isolation; Jahng et al., 2012; Gilabert-Juan et al., 2013) and performing qRT-PCR assessment of ErbB4 and NR2B mRNA levels (which are decreased in isolates; Gilabert-Juan et al., 2013; Loureiro et al., 2020) versus expression of mRNA encoding the GABA synthesis enzyme glutamic acid decarboxylase 67 (GAD67) and calcium binding protein parvalbumin (which are both unaffected by isolation; Begni et al., 2020). To maximise confidence in our key findings we demonstrate replication via qRT-PCR analysis of prefrontal cortical tissue from an independent cohort of animals.

## 2 Materials and methods

This research involved a total of 32 male Sprague-Dawley rats, who each represented a separate experimental unit. Sixteen animals were used for the primary study, which involved qRT-PCR to verify development of the isolation syndrome (because regulatory considerations prevented behavioural testing) and then the RNA-Seq that comprises the majority of the research presented here. The remaining 16 rats served as an independent validation cohort in a separate laboratory. They underwent behavioural testing to illustrate development of the isolation syndrome, followed by qRT-PCR replication of the key RNA-Seq findings.

### 2.1 Primary study

#### 2.1.1 Animals, housing and tissue collection

Rats in the primary study were obtained (Monash Animal Research Platform) on postnatal day (PND) 22–24. Half were randomly allocated (by drawing lots) to immediate social isolation (one per cage) in a barren environment, and the other half to conventional groups (four per cage) in an enriched environment, which represented the control condition. This cohort were housed in plastic cages (floor area 1500 cm^2^) with wire lids and all kept in the same holding room (21 ± 3 °C, 55 ± 15% relative humidity, 12h light-dark cycle; on 07:00h) to ensure isolates retained visual, auditory and olfactory contact with conspecifics. They were provided with bedding (Breeders Choice cat litter, FibreCycle; changed once weekly for isolates and twice weekly for group-housed counterparts due to the increased number of occupants) and had unrestricted access to food (Barastoc WEHI Mouse Breeder Cubes, Ridley AgriProducts) and water. Group-housed rats received environmental enrichment in the form of an empty tissue box and shredded paper. Body weight was measured at weekly intervals and the humane endpoint would have been euthanasia of any rat experiencing a decrease in body weight (up to a maximum permitted limit of −20%) and/or signs of poor body condition (e.g. piloerection, hunched posture, absence of grooming). In practice these endpoints were not encountered and all individuals showed a weekly increase in weight. Rats in the primary study were killed six weeks post-weaning (PND64-66) by terminal anaesthesia with isoflurane followed by cervical dislocation. This occurred throughout the working day, but care was taken to ensure a balanced mix of group-housed controls and social isolates were killed across morning and afternoon sessions to minimise any impact of circadian variations on the resulting data. Brains were rapidly removed and prefrontal cortical tissue (<30 mg) dissected into RNAse- and DNAse-free tubes, frozen on dry ice then stored at −80 °C until qRT-PCR validation of syndrome development or RNA-Seq (n = 4 group-housed controls and n = 4 isolates in each case, selected at random). Group sizes were chosen on the basis of previous DEG analyses that considered the impact of biological replicates (Zhang et al., 2014), since sample size calculations for RNA-Seq vary considerably depending on the tool and simulations show that most estimates are incorrect (Poplawski and Binder, 2018). Current group sizes are within the range of previous transcriptomic analysis into short-term social isolation followed by transient social play (n = 3; Alugubelly et al., 2019) and early life stress associated with provision of limited nesting material (n = 5; Green et al., 2021).

All procedures were conducted in accordance with the Australian Prevention of Cruelty to Animals Act (2004) and Australian National Health and Medical Research Code of Practice for the Care and Use of Animals for Experimental Purposes (2013), and approved by the Monash Institute of Pharmaceutical Sciences Animal Ethics Committee. Procedures were planned in order to minimise suffering and reduce the number of animals used, and are reported in accordance with the ARRIVE guidelines (Percie du Sert et al., 2020). Blinding of experimenters to group allocation throughout the six weeks of post-weaning housing was not possible due to the obvious visual difference between group and single housing. However, housing details were concealed throughout tissue processing and analysis.

#### 2.1.2 qRT-PCR validation of syndrome development

Total RNA was isolated using an Isolate II RNA Mini Kit (Bioline; BIO-52073) and the concentration determined using a NanoDrop® ND-1000 Spectrophotometer (ThermoFisher Scientific). Total RNA was reverse transcribed using a Tetro cDNA synthesis kit (Bioline; 65043) and Applied Biosystems 2720 Thermal Cycler. qRT-PCR was performed in triplicate with a LightCycler® 480 SYBR Green I Masterkit (Roche; 04707516001) and C1000 Touch™ Thermal Cycler. Forward and reverse primers for ErbB4 (TGGGATTAAAGAACCTGACCG, TGACACCAGAGTCATGTTGG), NR2B (GGGCTCATCTATGATAATGGCG, TGGTGACGATGGAAAAGATGTAC), GAD67 (ATACTTGGTGTGGCGTAGC, AGGAAAGCAGGTTCTTGGAG) and parvalbumin (CTGGACAAAGACAAAAGTGGC, GACAAGTCTCTGGCATCTGAG) genes of interest, as well as the housekeeping gene Ywhaz (GACGGAAGGTGCTGAGAAAA, GAAGACTTTGCTTTCTGGCTG), which showed stable expression between groups, were designed with the Integrated DNA Technologies (IDT) qPCR primer design tool (http://sg.idtdna.com/scitools/Applications/RealTimePCR/) using their pre-set parameters. Plates were read using the CFX384™ Real-Time System (Bio-Rad) with Bio-Rad CFX Manager 3.1 to give relative fold-changes in gene expression.

#### 2.1.3 RNA-Seq

Total RNA was extracted and purified using a Qiagen RNeasy® Mini Kit (Qiagen; 74104), with all samples processed on the same day. Following confirmation of suitable RNA quality (RNA Integrity Number >8) and quantity (>200 ng) using an AATI Fragment Analyzer and Invitrogen Qubit assay, purified samples were submitted to Micromon Genomics, Monash University, Australia for cDNA library preparation according to manufacturer's instructions (Illumina TruSeq Stranded mRNA kit) and subsequent multiplex sequencing using Illumina NextSeq 500.

Single-end 75 base pair sequence reads were subject to quality control with FastQC v0.11.9 (https://www.bioinformatics.babraham.ac.uk/projects/fastqc/; Wingett and Andrews, 2018). Reads were mapped to the Ensembl *Rattus norvegicus* reference genome (Rnor_6.0, INSDC Assembly; July 2014) using STAR aligner v2.6.1 (https://github.com/alexdobin/STAR; Dobin and Gingeras, 2015). Data manipulation, further quality control and read summarisation were performed using Samtools v1.9 (http://www.htslib.org/; Li et al., 2009), Picard v2.18.22 (http://broadinstitute.github.io/picard/), QualiMap v2.2.1 (http://qualimap.bioinfo.cipf.es/; Okonechnikov et al., 2016) and FeatureCounts v1.6.3 (http://bioinf.wehi.edu.au/featureCounts/; Liao et al., 2014). A single summary report was created using MultiQC v1.7 (https://multiqc.info/; Ewels et al., 2016) to facilitate quality control.

Differences in gene expression between group-housed control and isolation-reared rats were determined with Degust v3.1.0 (http://degust.erc.monash.edu/) using the voom/limma function, which is recommended for the current sample size (Schurch et al., 2016). P values were corrected for multiple testing and genes were considered differentially expressed if the adjusted P value was <0.05. Biological insight into DEGs was obtained by mapping to functional data sources using g:Profiler vE98.EG45 (https://biit.cs.ut.ee/gprofiler/gost; Raudvere et al., 2019), with a false discovery rate (FDR) cutoff of 0.05, followed by enrichment analysis using the Gene Ontology (GO) database. Isolation-induced DEGs were also uploaded to Ingenuity Pathway Analysis (IPA) software (QIAGEN Digital Insights; Winter Release, 2022) to enable unbiased insight into altered canonical signalling pathways or upstream regulators that exceeded a threshold of -log(P value) ≥1.3 and z-score ≤ -2 or ≥2. Lastly DEGs and enriched biological functions identified in the PFC of isolation-reared rats were compared against those in post-mortem frontal cortical tissue from 12 separate published or open access RNA-Seq datasets (Supplementary Table 1) involving a total of 728 patients (schizophrenia n = 555, BD n = 7, MDD n = 56 or ASD n = 110) and 943 healthy controls. The drug-gene interaction database (DGIdb v4.2.0; https://www.dgidb.org/) was used to identify shared DEGs that represent druggable targets and to provide a shortlist of relevant drug-gene interactions (Freshour et al., 2021).

### 2.2 Independent validation study

#### 2.2.1 Animals and housing

To maximise confidence in the reproducibility of our RNA-Seq data, the validation study was conducted in a separate laboratory. Most procedures were as described in Section 2.1.1., but the geographical separation necessitated different suppliers for the animals themselves as well as cages, diet and bedding. We also permitted some minor variations in husbandry procedures, to allow each institution to follow usual local practices. Rats in the validation study were obtained (Envigo, UK) on PND21. Cages were 1632 cm^2^ for groups and 1050 cm^2^ for isolates. They contained sawdust bedding (changed once weekly) without environmental enrichment (for consistency with previous in-house studies; e.g. Fone and Porkess, 2008; Shortall et al., 2018; Shortall et al., 2020) and were housed within a scantainer ventilated cabinet (Scanbur) to limit human exposure to animal allergens. Environmental conditions were 21 ± 2 °C, 55 ± 10% relative humidity and a 12h light-dark cycle with a 15 min gradual change over (half the lights on at 06:45h, all the lights on at 07.00h). Diet was 2918 Teklad Irradiated Global 18% Protein Rodent Diet (Inotiv). Procedures were conducted in accordance with the Animals (Scientific Procedures) Act, 1986, with approval from the University of Nottingham Animal Welfare and Ethical Review Body (AWERB). Group sizes (n = 8 group-housed controls and n = 8 isolates) were based on power calculations for relevant behavioural tests.

#### 2.2.2 Behavioural validation of the isolation syndrome

Commonly-reported behavioural changes in isolation-reared male Sprague-Dawley rats include increased anxiety (shown by reduced duration or entries into the central zone of an open field apparatus, e.g. Lukkes et al., 2009; Sciolino et al., 2010; Meng et al., 2010; Sun et al., 2017; Cao et al., 2021) and cognitive deficits (shown by impaired novel object recognition, e.g. Zamberletti et al., 2012; Möller et al., 2013; Uys et al., 2016; Atmore et al., 2020; Shangase et al., 2024). We therefore selected these behavioural tests for our own validation of the isolation syndrome. Exploratory activity in an open field was assessed on PND63, by placing individual rats into a brightly lit circular arena (75 cm diameter with 45 cm high walls, 395Lux at floor level) for 10min and tracking time spent in the outer (15 cm from the wall), middle (15–30 cm from the wall) and very central (>30 cm from the wall) zones (Noldus Ethovision XT v17 software). Rats were all consistently placed into the arena in the outer zone, facing towards the wall. Later the same day rats were habituated to one of eight identical Perspex arenas (39 × 23.5 × 24.5 cm; average light intensity at floor level) for 1h, in preparation for the novel object recognition test. The following day (PND64) they received a further 3min habituation to their designated arena, then two consecutive 3 min object exploration trials separated by 2h. In the familiarization trial, rats encountered two identical plastic bottles covered in white tape. For the choice trial, one was randomly replaced with a novel object (identical size and shape, with three stripes of black tape). Object exploration (sniffing, licking, chewing, or having moving vibrissae while directing the nose towards and ≤1 cm) was manually timed using stopwatches. The 2h inter-trial interval was chosen for consistency with numerous previous studies in which group-housed controls had intact memory but isolates did not (e.g. King et al., 2009; Shortall et al., 2018; Shortall et al., 2020). All behavioural apparatus was cleaned with 20% ethanol between trials.

#### 2.2.3 Tissue collection and qRT-PCR

Rats in the validation study were killed immediately after novel object recognition testing (PND64) by concussion followed by decapitation. Brains were rapidly removed onto a refrigerated table (4 °C) and prefrontal cortical tissue (<50 mg) dissected into RNAse- and DNAse-free tubes, containing 300 μL RNAlater™ (Sigma-Aldrich). Samples were stored at 4 °C for 3d then at −20 °C until qRT-PCR. Total RNA was isolated using TRIzol® Reagent (ThermoFisher Scientific) then resuspended in RNase-free water and stored at −70 °C. The RNA concentration was determined using a NanoDrop® 2000c UV–Vis Spectrophotometer (ThermoFisher Scientific). cDNA was synthesised from 200 ng total RNA using SuperScript™ IV reverse transcriptase and Oligo(dT)20 primer (Invitrogen). qRT-PCR was performed using a PowerUp™ SYBR™ Green master mix kit (Applied Biosystems) and an Applied Biosystems QuantStudio5™ thermocycler, which was used in standard mode with cycling parameters recommended by Applied Biosystems. Data were acquired using QuantStudio™ design and analysis software v1.5.2. Each sample was run in duplicate using forward and reverse primers for the genes of interest, which were *Hrh3* (TGCTCAACCTCGCCATCTC, GGAGGCACACAGTAGGTAGTC), *Snca* (TCAGCCCAGAGCCTTTCAC, CTTCCGCAGAACTCTGATTCC), *Sod1* (CTTGGCTTGTGGTGTGATTGG, CAGTTTAGCAGGACAGCAGATG), *Chrm4* (ATCGGCCGAAGTGCATAGAG, GCGGAAAGCACCGATTACAG), *Klf2* (GTGACCGTGGCAATCTGTATG, GCCACAGCACTACCATGATTC), *Lrrk2* (CACTTCTGCCCAGGTCTTTG, TGAGGTTTGTAGCAGCTATGC), *Nr4a1* (GTAGTGTGCGAGAAGGATTGC, GCTTGGATACAGGGCATCTC), *Nr4a3* (CTGTGACTCTCCCCCAATCC, CAGTGGGCTTTGGGTTCTG) and *Prkca* (GGAGGATCGACTGGGAGAAG, TCCTTTGCCGCACACTTTG), and the housekeepers *18s* (CGGACAGGATTGACAGATTG, CAAATCGCTCCACCAACTAA), *β-Actin* (CTAAGGCCAACCGTGAAAAGA, ACAACACAGCCTGGATGGCTA) and *B2M* (GTCTTTCTACATCCTGGCTCACA, GACGGTTTTGGGCTCCTTCA). Primers were designed with the ThermoFisher Scientific OligoPerfect primer design tool. ROX™ (Fisher Scientific) was used as a passive reference for normalising for non-PCR related fluorescence variations, and was incorporated by the software to calculate Ct values. Data were normalised using the comparative Ct method (2^−ΔΔCt^) with the geometric mean of the housekeeping genes used as the endogenous control and mean group-housed data as the calibrator, and expressed as fold-change.

### 2.3 Statistical analysis

No exclusion criteria were set and data from all animals were included in the analyses, which were planned before the study took place (although not formally registered). Body weight, behavioural and qRT-PCR data were plotted and analysed using GraphPad Prism (v10.3.0). Normality was confirmed using the Kolmogorov-Smirnov test prior to parametric analyses. Body weight, open field and novel object recognition data were analysed by two-way repeated measures ANOVA (with time, zone or object as within- and housing as between-subjects factor). qRT-PCR data were analysed by Mann Whitney *U* test or standard two-way ANOVA (with gene and housing as between-subject factors). ANOVAs were followed by Sidak's post-hoc test. P < 0.05 was considered statistically significant. Data analysed with parametric tests are presented as mean ± standard error of the mean (SEM) and those analysed with non-parametric tests as median ± interquartile range (IQR).

## 3 Results

### 3.1 Primary study

#### 3.1.1 Confirmation of the isolation syndrome

**Fig. 1:**
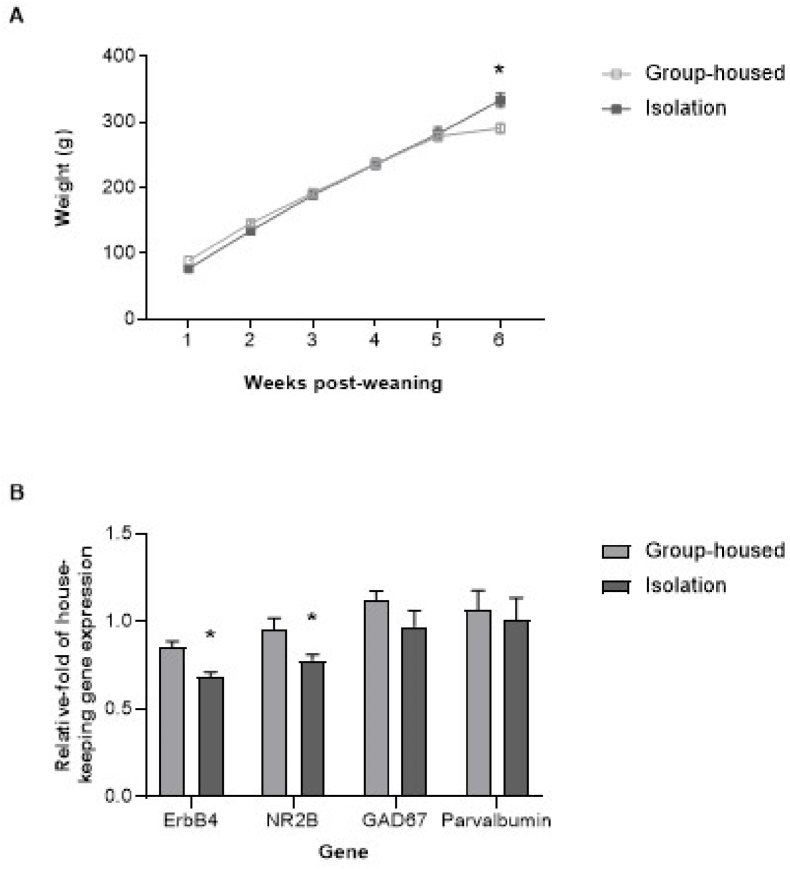
Confirmation of isolation syndrome development prior to RNA sequencing. Mean ± SEM (**A**) body weight of male Sprague-Dawley rats housed in social groups (4 per cage) or isolation (1 per cage) from weaning on postnatal day (PND) 22–24 until tissue collection on PND64-66 (n = 8 per housing condition), and (**B**) prefrontal cortical expression of four mRNAs selected on the basis of reported down-regulation (ErbB4, NR2B) or stability (GAD67, parvalbumin) following isolation rearing, presented as relative-fold of the Ywhaz housekeeping control gene (n = 4 per housing condition, selected at random). ∗P < 0.05 versus group-housed controls (two-way repeated measures ANOVA for body weight data in **A**, and two-way ANOVA for expression data in **B**, followed by Sidak's post-hoc test in each case).

Body weights changed over time (F_(5,62)_ = 790.3, P < 0.0001) and demonstrated a time × housing interaction (F_(5,62)_ = 5.280, P = 0.0004), with isolates being 15% heavier than group-housed controls by six weeks post-weaning (Fig. 1A). There was a main effect of housing on frontal cortical levels of ErbB4 and NR2B mRNA (F_(1,12)_ = 17.92, P = 0.0012), which were down-regulated by 20% and 18% respectively in isolates. In contrast, levels of GAD67 and parvalbumin mRNA were unaffected by housing (F_(1,12)_ = 1.184, P = 0.2980) and not significantly down-regulated in isolates (Fig. 1B). These findings match previous reports following isolation rearing (Shelley, 1965; Simpson et al., 2012; Gilabert-Juan et al., 2013; Begni et al., 2020; Loureiro et al., 2020; Kaneda et al., 2021; Tzeng et al., 2023). In addition, subjective observations during routine husbandry procedures revealed pronounced isolation-induced behavioural changes in the current cohort that are in line with those we (unpublished observations) and others (e.g. Tulogdi et al., 2014) have observed. These include frequent adoption of a defensive upright posture in response to non-threatening movement of rats in adjacent cages or to being approached by the handler. Isolates also exhibited increased locomotion and vocalisation in response to routine handling. These features are common to our previous cohorts, where formal behavioural assessment (by observers unaware of housing allocation) revealed robust locomotor and cognitive changes (e.g. Jones et al., 2011; Dunphy-Doherty et al., 2018; Shortall et al., 2018; Shortall et al., 2020). We therefore progressed tissue from this cohort of animals to planned RNA-Seq analyses.

#### 3.1.2 Differentially expressed genes

**Fig. 2:**
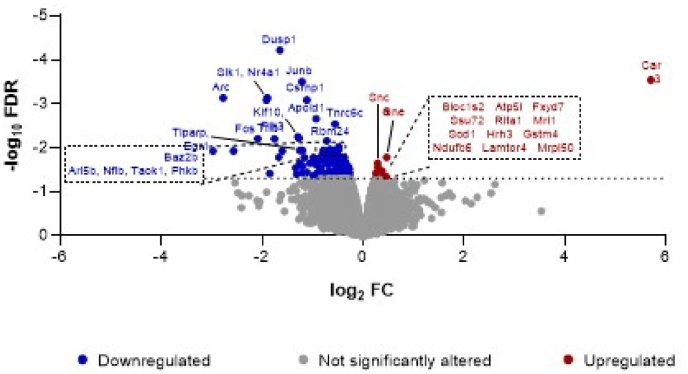
Volcano plot of statistical significance (log_10_ false discovery rate; log_10_ FDR) versus magnitude of change (log_2_ fold-change; log_2_ FC) for differentially expressed genes (DEGs) in the rat prefrontal cortex following isolation rearing. Male Sprague-Dawley rats were housed in social groups or isolation from weaning on postnatal day (PND) 22–24 until tissue collection on PND64-66 (n = 4 per housing condition). 183 genes were differentially expressed in isolates, of which 15 were up-regulated (red circles and gene symbols; log_2_ FC > 0, P < 0.05) and 168 were down-regulated (blue circles with gene symbols also shown for the 20 most significantly down-regulated; log_2_ FC < 0, P < 0.05). Full names are provided in Table 1, where the level of significance is also reported. (For interpretation of the references to colour in this figure legend, the reader is referred to the Web version of this article.)

A total of 183 DEGs with a diverse array of functions were detected in isolates compared to group-housed controls, of which 15 were up-regulated (log_2_ fold-change: 0.26 to 5.70) and 168 down-regulated (log_2_ fold-change: 2.97 to −0.25; Fig. 2). 98.9% of these DEGs were classified as protein coding, with the only exceptions being *Arid5b* and *Rc3h1* that were identified as processed transcripts. The 15 up-regulated and the top 20 down-regulated genes (arranged by adjusted P value) are presented in Table 1 and an alphabetical list of all 183 DEGs is provided in Supplementary Table 2. In terms of fold-change the most highly up-regulated was *Car3* and the most highly down-regulated were *Arc*, *Dnah17*, *Egr2* and *Fos*, followed by *Asmt*, *Dusp1*, *Klhl15*, *Nr4a1*, *Nr4a3*, *Sik1*, *Sntb2*, *Snurf* and *Trib1* (log_2_ fold-change ≤ -1.3). We note that DEGs identified by RNA-Seq matched our preliminary qRT-PCR analyses of isolation syndrome development in this cohort, in that when the four relevant transcripts were considered separately from the remaining >14,000 members of our RNA-Seq dataset, both *ErbB4* and *Grin2b* (which encodes the NMDA receptor NR2B subunit) were significantly down-regulated (P = 0.0092 and P = 0.0005, respectively) whereas *GAD1* and *Pvalb* (which encode GAD67 and parvalbumin) were not. When the RNA-Seq data were analysed as a whole, down-regulation of *Grin2b* survived FDR correction (P = 0.0464; Supplementary Table 2) but down-regulation of *ErbB4* fell outside the cutoff (P = 0.1191). These findings maximise confidence in the data obtained with our RNA-Seq workflows, which appear to be more stringent than those obtained with qRT-PCR.

**Table 1:**
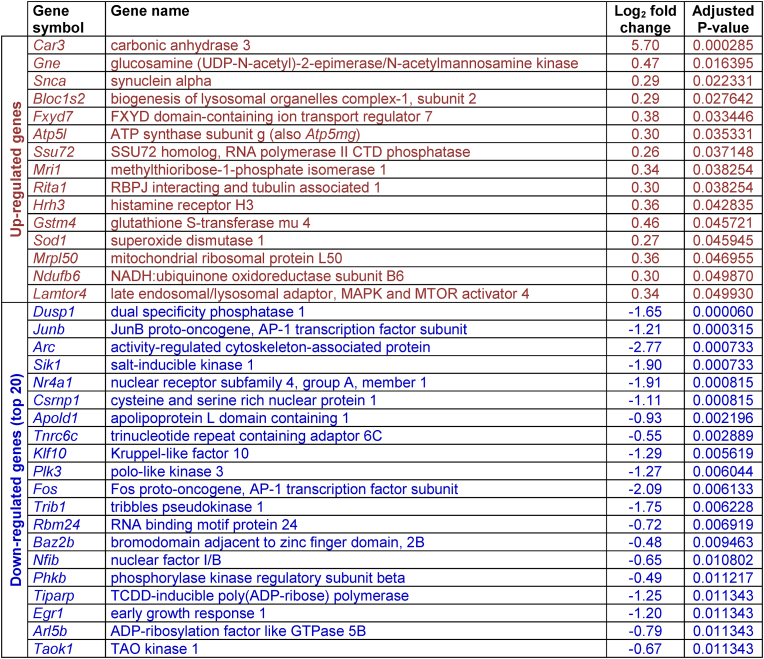
Differentially expressed genes (DEGs) in the rat prefrontal cortex following isolation rearing. Male Sprague-Dawley rats were housed in social groups or isolation from weaning on postnatal day (PND) 22–24 until tissue collection on PND64-66 (n = 4 per housing condition). Details (gene symbol, name, log_2_ fold-change and adjusted P value) are provided for all 15 up-regulated DEGs (red) and the top 20 down-regulated DEGs (blue), sorted in descending order by adjusted P value. A full alphabetical list of all 183 DEGs is provided in Supplementary Table 2.

#### 3.1.3 Functional enrichment and Ingenuity Pathway Analysis

**Fig. 3:**
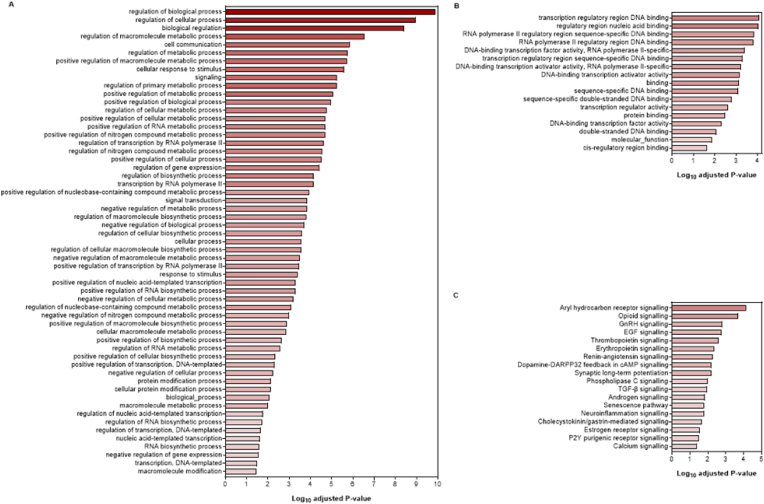
Gene Ontology (GO) enrichment analysis and Ingenuity Pathway Analysis (IPA) of differentially expressed genes (DEGs) in the rat prefrontal cortex following isolation rearing. Male Sprague-Dawley rats were housed in social groups or isolation from weaning on postnatal day (PND) 22–24 until tissue collection on PND64-66 (n = 4 per housing condition). Isolation-induced DEGs are implicated in significantly (P < 0.05) enriched (**A**) GO biological processes, (**B**) GO molecular functions and (**C**) IPA signalling pathways, with increasing log_10_ adjusted P values and higher colour intensities indicating higher levels of enrichment. (For interpretation of the references to colour in this figure legend, the reader is referred to the Web version of this article.)

Functional analyses of isolation-induced DEGs revealed enrichment in each of the three GO categories (57 biological processes, 17 molecular functions and a single cellular component). The enriched biological processes were relevant to cellular, metabolic and biosynthetic processes, cellular communication and cellular response to stimuli (Fig. 3A). Enriched molecular functions related mainly to nucleic acid or protein binding and regulation of transcription (Fig. 3B). The only enriched cellular component was the nucleus (adjusted P = 0.0356), although this does not mean all DEGs were confined to that location.

**Fig. 4:**
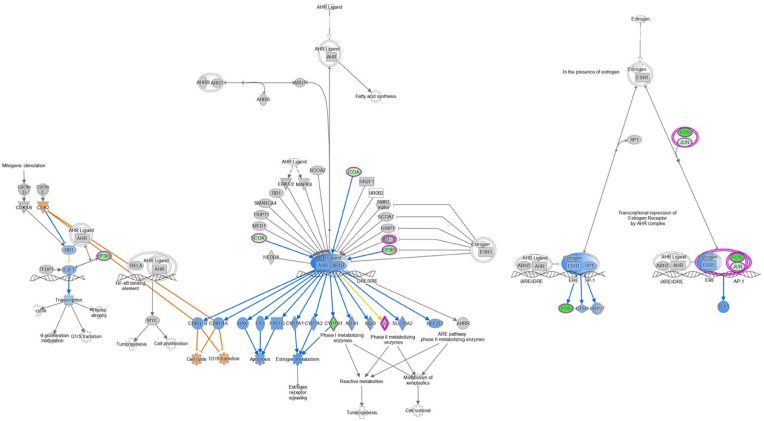
Ingenuity Pathway Analysis (IPA) of differentially expressed genes (DEGs) implicated in the aryl hydrocarbon receptor signalling pathway. Green shapes with pink borders indicate genes that were down-regulated in the rat prefrontal cortex following isolation rearing; ovals represent those encoding transcription regulators and diamonds represent those encoding enzymes. Blue indicates predicted inhibition, orange predicted activation and yellow predictions inconsistent with known expression of downstream molecules. Full names of the DEGs are provided in Supplementary Table 2. (For interpretation of the references to colour in this figure legend, the reader is referred to the Web version of this article.)

IPA revealed significant overlap of our DEGs with 18 signalling pathways. These ranged from aryl hydrocarbon receptor, opioid, gonadotropin-releasing hormone (GnRH) and epidermal growth factor (EGF) signalling to dopamine- and cAMP-regulated phosphoprotein 32 kDa (DARPP-32) feedback, long-term potentiation (LTP) and neuroinflammation (Fig. 3C). In each case z-scores were negative, indicating predicted inhibition. A total of 27 DEGs were implicated in the inhibited pathways, although the number of DEGs in each pathway was relatively low (average 6 per pathway, range 4–10; Table 2). Approximately 50% of DEGs were linked to multiple pathways, with the most promiscuous being *Adcy1*, *Cacna1e*, *Ep300*, *Grin2b* and to an even greater extent (≥10 pathways) *Fos*, *Itpr1*, *Jun* and *Prkca*. In contrast, DEGs like *Cyp1b1*, *Gstm4*, *Nfib* and *Nfix* were restricted to aryl hydrocarbon receptor signalling as their single implicated pathway (Fig. 4), with *Bhlhe40*, *Gadd45b* and *Hipk2* showing similar restriction to senescence. Additional regulatory analyses using IPA implicated upstream PKC inhibition as a potential driver of *Egr1*, *Egr2*, *Fos*, *Jun* and *Per1* down-regulation (z-score −2.216). Other weaker drivers included calcineurin expression for *Arc*, *Egr1*, *Egr2*, *Fos*, *Itpr1*, *Nr4a1* and *Nr4a3* down-regulation (z-score −1.044), as well as TNFα for *Cyp1b1*, *Egr1*, *Fos*, *Nr4a3* down-regulation and *Sod1* up-regulation (z-score −0.73).

**Table 2:** Ingenuity Pathway Analysis (IPA) of differentially expressed genes (DEGs) in the rat prefrontal cortex following isolation rearing. Male Sprague-Dawley rats were housed in social groups or isolation from weaning on postnatal day (PND) 22–24 until tissue collection on PND64-66 (n = 4 per housing condition). IPA signalling pathways are arranged in descending order by negative z-score, which reflects the extent of pathway inhibition, then by adjusted P value (shown in Fig. 3C). Up-regulated genes are underlined and the remainder are down-regulated. A full alphabetical list of all 183 DEGs is provided in Supplementary Table 2.

IPA canonical pathway	z-score	DEGs
Opioid signalling	−2.65	*Adcy1*, *Cacna1e*, *Egr4*, *Ep300*, *Fos*, *Fosb*, *Grin2b*, *Itpr1*, *Kcnj6*, *Prkca*
Estrogen receptor signalling	−2.65	*Adcy1*, *Cacna1e*, *Ep300*, *Fos*, *Jun*, *Ncoa3*, *Ndufb6*, *Prkca*
Aryl hydrocarbon receptor signalling	−2.45	*Cypb1*, *Ep300*, *Fos*, *Gstm4*, *Jun*, *Ncoa3*, *Nfib*, *Nfix*
GnRH signalling	−2.45	*Adcy1*, *Cacna1e*, *Egr1*, *Fos*, *Itpr1*, *Jun*, *Prkca*
Erythropoietin signalling	−2.45	*Fos*, *Irs2*, *Itpr1*, *Jun*, *Prkca*, *Xiap*
Senescence pathway	−2.45	*Acvr1c*, *Bhlhe40*, *Cacna1e*, *Ep300*, *Gadd45b*, *Hipk2*, *Jun*
Neuroinflammation signalling	−2.45	*Acv1rc*, *Fos*, *Grin2b*, *Jun*, *Kcnj6*, *Snca*, *Xiap*
Dopamine-DARPP32 feedback in cAMP signalling	−2.24	*Adcy1*, *Cacna1e*, *Grin2b*, *Itpr1*, *Kcnj6*, *Prkca*
Synaptic long-term potentiation	−2.24	*Adcy1*, *Ep300*, *Grin2b*, *Itpr1*, *Prkca*
EGF signalling	−2	*Fos*, *Itpr1*, *Jun*, *Prkca*
Thrombopoietin signalling	−2	*Fos*, *Irs2*, *Jun*, *Prkca*
Renin-angiotensin signalling	−2	*Adcy1*, *Fos*, *Itpr1*, *Jun*, *Prkca*
Phospholipase C signalling	−2	*Adcy1*, *Arhgef12*, *Ep300*, *Hdac4*, *Itgb8*, *Itpr1*, *Prkca*
TGF-β signalling	−2	*Acvr1*, *Ep300*, *Fos*, *Jun*
Androgen signalling	−2	*Cacna1e*, *Ep300*, *Itpr1*, *Jun*, *Prkca*
Cholecystokinin/gastrin-mediated signalling	−2	*Fos*, *Itpr1*, *Jun*, *Prkca*
P2Y purigenic receptor signalling	−2	*Adcy1*, *Fos*, *Jun*, *Prkca*
Calcium signalling	−2	*Cacna1e*, *Ep300*, *Grin2b*, *Hdac4*, *Itpr1*

#### 3.1.4 Overlap of isolation-induced changes with those in patient populations

**Fig. 5:**
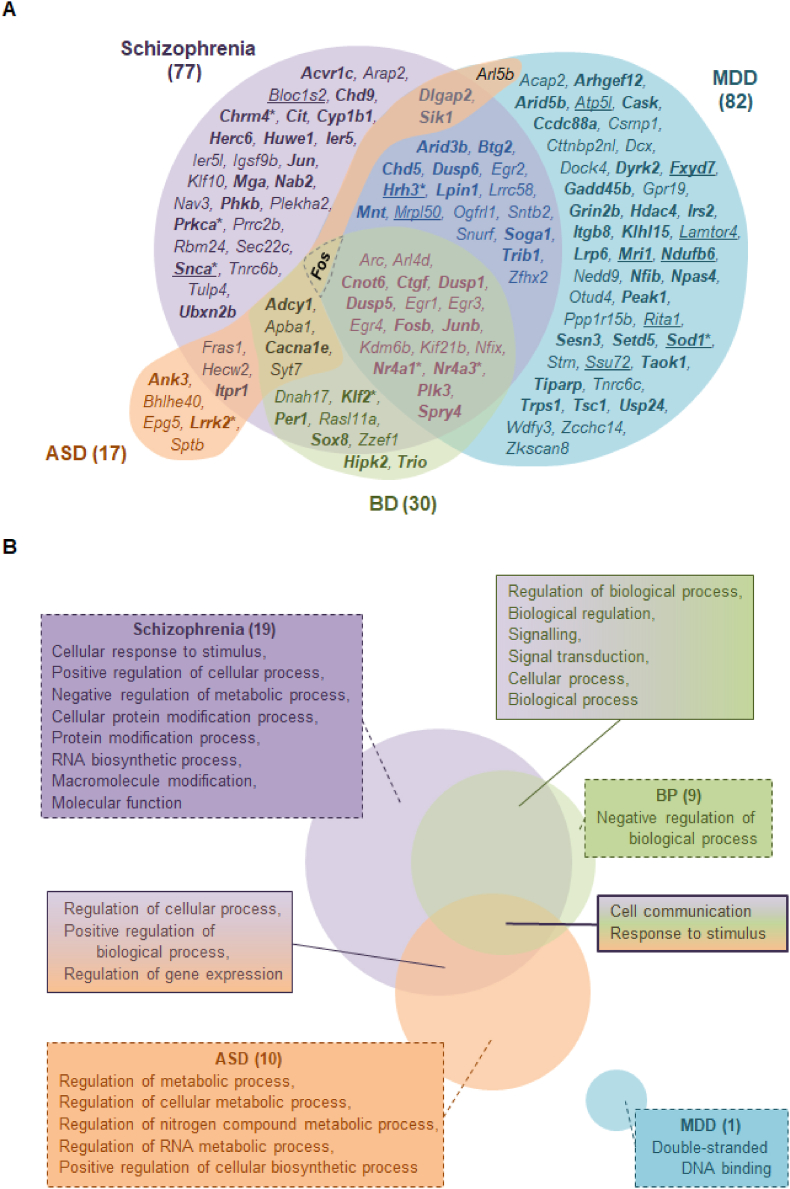
Overlap of differentially expressed genes (DEGs) and enriched Gene Ontology (GO) terms in the rat prefrontal cortex following isolation rearing with those in patient populations. Venn diagrams illustrating overlap of isolation-induced (**A**) DEGs and (**B**) GO terms with those reported in frontal cortical tissue from patients with schizophrenia (purple), major depressive disorder (MDD; blue), bipolar disorder (BD; green) or autistic spectrum disorder (ASD; orange). Male Sprague-Dawley rats were housed in social groups or isolation from weaning on postnatal day (PND) 22–24 until tissue collection on PND64-66 (n = 4 per housing condition). Further details on the patient cohorts are provided in Supplementary Table 1 and full names of the DEGs are provided in Table 1 and/or Supplementary Table 2, where the level of significance is also reported. In (**A**) up-regulated genes are underlined and the remainder are down-regulated. Bold indicates druggable targets identified using the drug-gene interaction database (DGIdb) and asterisks indicate availability of pharmacological agents predicted to oppose isolation- and disease-related changes. In (**B**) two- and three-tone shading indicates enriched terms that are shared between isolation rearing and multiple patient populations. (For interpretation of the references to colour in this figure legend, the reader is referred to the Web version of this article.)

One hundred and twenty-eight (70%) of the isolation-induced DEGs identified here (12 up-regulated, 116 down-regulated) mirrored those in PFC tissue from patients with psychosocial stress-related mental illnesses and/or neurodevelopmental conditions featuring social deficits (Fig. 5A). The overlap was greatest for schizophrenia (42%) and MDD (45%) and more modest for BD (16%) and ASD (9%). 43% of isolation-induced DEGs appeared to be shared with a single disorder but numerous down-regulated DEGs were shared across conditions. These include 14% common to two of the four considered disorders and 13% common to isolation and three disorders (the latter pool being *Adcy1*, *Apba1*, *Arc*, *Arl4d*, *Cacna1e*, *Cnot6*, *Ctfg*, *Dlgap2*, *Dusp* and *Egr* family members, *Fosb*, *Junb*, *Kdm6*, *Kif21b*, *Nfix*, *Nr4a1*, *Nr4a3*, *Plk3*, *Sik1*, *Spry4* and *Syt7*). Only a single gene, *Fos* (representing 0.5% of the isolation-induced changes), showed common down-regulation across all conditions.

Twenty-six (35%) of the enriched GO biological processes and molecular functions identified here overlapped with those reported in patients with schizophrenia (26%), BD (12%) or ASD (14%), but overlap with MDD was limited to a single term (1%). However, this may be due to the smaller number of human datasets available for comparison of GO enrichment compared to DEG analyses. Enrichment for cell communication and response to stimulus were shared between isolation and the other three disorders, and terms relating to signalling, signal transduction and regulation of biological processes were shared between isolation and various pairs of disorders (Fig. 5B).

Seventy-one of the DEGs shared between isolation-reared rats and the selected patient populations encode proteins identified as druggable targets by the DGIdb. These include G protein-coupled receptors (e.g. *Chrm4*, *Hrh3*), serine/threonine kinase receptors (e.g. *Acvr1c*), ion channels or their subunits (e.g. *Cacna1e*, *Grin2b*, *Itpr1*), proteins that regulate ion channel function (e.g. *Ank3*) and nuclear hormone receptors (e.g. *Chd9*, *Nr4a1*, *Nr4a3*). There are also various enzymes (e.g. *Adcy1*, *Arhgef12*, *Cyp1b1*, *Hdac4*) including serine- and threonine-specific protein kinases (e.g. *Hipk2*, *Prkca*), as well as transcription factor subunits (e.g. *Fos*, *Fosb*, *Jun*, *Junb*) and transcription repressors (e.g. *Nab2*; Fig. 5A, names in bold). Interestingly, there are antagonists or inhibitors for the products of three DEGs that are up-regulated in isolation-reared rats and patients with schizophrenia (*Snca*), depression (*Sod1*) or both (*Hrh3*). There are also agonists or activators for products of six DEGs that are down-regulated in ASD (*Lrrk2*), schizophrenia (*Chrm4*, *Prkca*), schizophrenia and BP (*Klf2*) or schizophrenia, BP and MDD (*Nr4a1* and *Nr4a3*; Table 3). These nine DEGs were selected for independent qRT-PCR validation, using prefrontal cortical tissue from a separate cohort of animals.

**Table 3:**
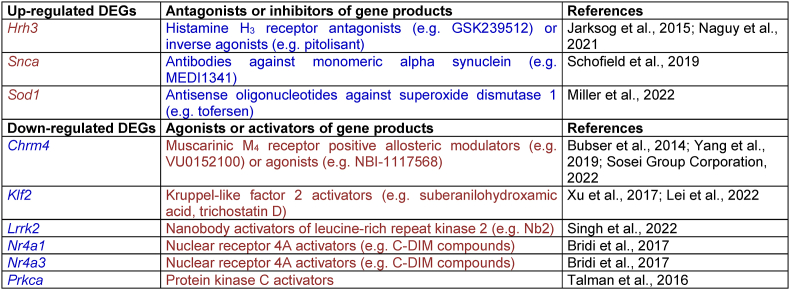
Existing pharmacological agents predicted to oppose the consequences of isolation- and disease-related changes in gene expression. Antagonists or inhibitors (blue) would be required to normalise the consequences of up-regulated DEGs (red), while agonists or activators (red) wold be required to normalise the consequences of down-regulated DEGs (blue). Some of the suggested modulators for differentially expressed genes (DEGs) have reached clinical evaluation for psychiatric disorders, albeit without patient stratification according to life history of stress exposure (e.g. H_3_ and M_4_ receptor ligands). Other approaches are at earlier stages of development, mainly in the context of neurodegeneration. Changes to six of the nine DEGs (67%) were replicated by qRT-PCR analysis of prefrontal cortical tissue from an independent cohort of animals (Fig. 6D) and findings for two further DEGs were very close to significance (P = 0.0571–0.0637) (Bubser et al., 2014, Schofield et al., 2019).

### 3.2 Independent validation study

#### 3.2.1 Behavioural confirmation of the isolation syndrome

**Fig. 6:**
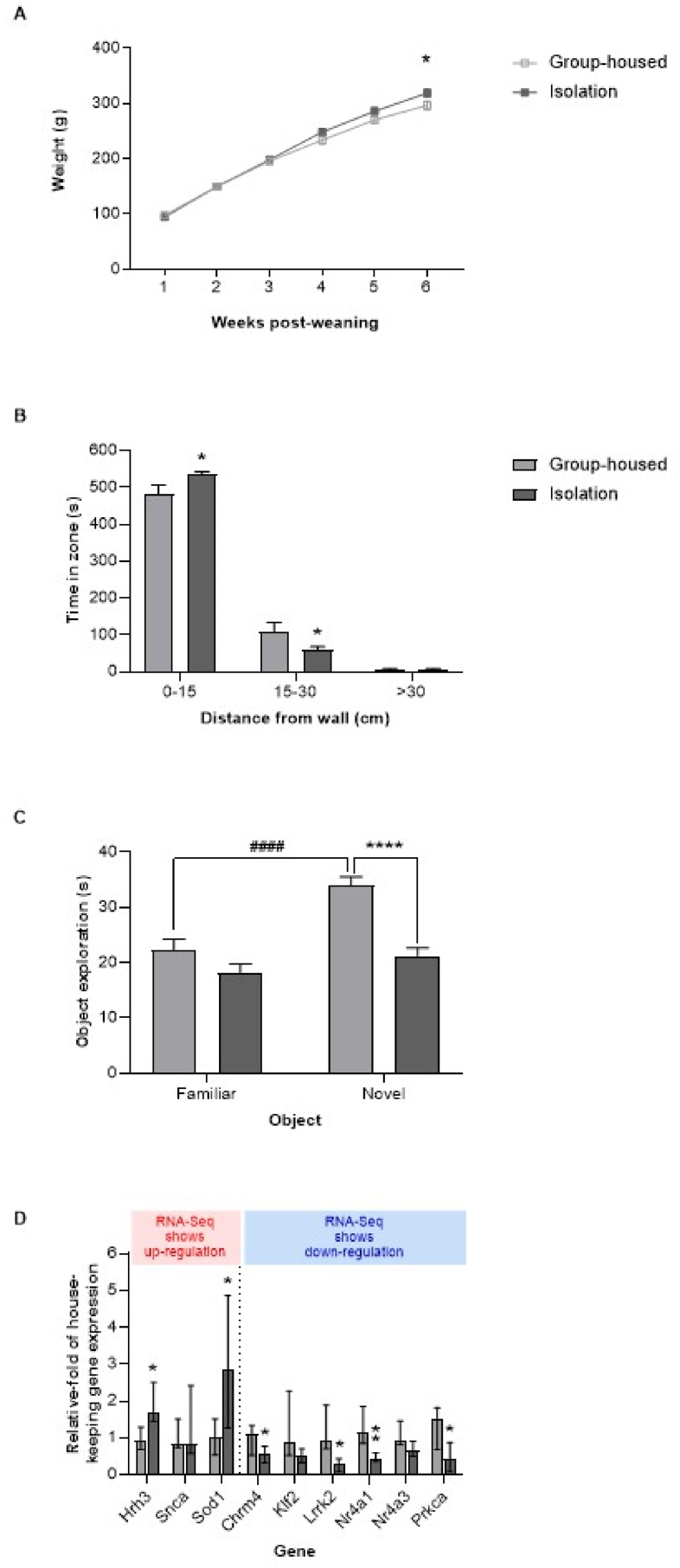
Replication of key RNA-Seq findings via qRT-PCR analysis of prefrontal cortical tissue from an independent cohort of isolation-reared rats. Male Sprague-Dawley rats were housed in social groups (4 per cage) or isolation (1 per cage) from weaning on postnatal day (PND) 21 until behavioural assessment in the open field test on PND63 then novel object recognition and tissue collection on PND64 (n = 8 per housing condition). Mean ± SEM (**A**) body weight or time spent (**B**) in central and peripheral zones of the open field and (**C**) exploring novel versus familiar objects during the novel object recognition choice trial, together with (**D**) median ± IQR prefrontal cortical expression of selected mRNAs highlighted by our RNA-Seq as overlapping with changes in one or more patient populations, and whose consequences should be opposed by currently available pharmacological agents. ∗P < 0.05, ∗∗P < 0.01, ∗∗∗∗P < 0.0001 isolates versus group-housed controls (two-way repeated measures ANOVA Sidak's post-hoc for data in **A**-**C**, and Mann-Whitney *U* test for data in **D**) and ####P < 0.0001 novel versus familiar following the same housing conditions (two-way repeated measures ANOVA Sidak's post-hoc).

Similarly to the primary study, body weights changed over time (F_(5,70)_ = 1711, P < 0.0001) and demonstrated a time × housing interaction (F_(5,70)_ = 7.211, P < 0.0001), with isolates being 8% heavier than group-housed controls by six weeks post-weaning (Fig. 6A). Exploratory activity in the open field showed a main effect of zone (F_(2,42)_ = 696.7, P < 0.0001) and a zone × housing interaction (F_(2,42)_ = 6.074, P = 0.0048). Compared to group-housed controls, isolates spent more time in the outer zone (i.e. the 15 cm closest to the walls) and less time in the middle zone (i.e. 15-30 cm from the walls; Fig. 6B), which is indicative of isolation-induced increases in anxiety. Time in the small very central zone (>30 cm from the walls) was low in both groups, consistent with this being the most aversive region. In the novel object recognition test both groups spent an equal amount of time exploring the two identical objects during the familiarization trial, such that there was no main effect of housing (F_(1,14)_ = 0.000, P > 0.9999) or object (F_(1,14)_ = 0.0519, P = 0.8232), no housing × object interaction (F_(1,14)_ = 2.080, P = 0.1713) and no post-hoc difference in exploration of object 1 versus object 2 (P > 0.05). This is consistent with both groups having an equivalent learning opportunity. During the choice trial 2h later there were main effects of housing (F_(1,14)_ = 20.85, P = 0.0004) and object (F_(1,14)_ = 26.29, P = 0.0002), plus a housing × object interaction (F_(1,14)_ = 9.250, P = 0.0088). Group-housed controls spent significantly longer exploring the novel than the familiar object, which indicates intact memory. In contrast the isolates spent an equivalent length of time exploring their two objects, with exploration of the novel object being lower than in control animals, which all indicates impaired memory (Fig. 6C). Taken together these findings confirm robust development of the isolation syndrome in this cohort and we therefore progressed tissue to planned qRT-PCR analyses.

#### 3.2.2 qRT-PCR

Our investigations focussed on nine DEGs highlighted by RNA-Seq that overlap with changes in one or more patient populations, and whose consequences should be opposed by currently available pharmacological agents (Table 3). qRT-PCR analyses of our independent cohort replicated significant upregulation of *Sod1* and *Hrh3* and significant downregulation of *Chrm4*, *Lrrk2*, *Nr4a1* and *Prkca* (Fig. 6D), while the effects on *Klf2* and *Nr4a3* both approached significance (P = 0.0571 and 0.0637 respectively).

## 4 Discussion

RNA-Seq has been used to profile transcriptome-wide changes in gene expression in numerous pathologies (from cancers and cardiovascular diseases to neuropsychiatric illnesses) to elucidate underlying mechanisms and identify new targets for therapeutic intervention (e.g. Li and Teng, 2016; Nakagawa and Fujita, 2018; Lu and Thum, 2019). The same approach has been applied to disease models, where it also provides insights into face validity. Although others have examined brain regional consequences of various risk factors for mental illness (e.g. Smagin et al., 2016; Tomas-Roig et al., 2016; Zhang et al., 2016; McCann et al., 2019; Reshetnikov et al., 2021) to our knowledge we are the first to use RNA-Seq for transcriptome-wide analysis of PFC gene expression following post-weaning isolation rearing of rats. This manipulation mimics chronic psychosocial stress throughout adolescent neurodevelopment (Heidbreder et al., 2000; Fone and Porkess, 2008), which has become more pertinent due to the SARS-CoV-2 pandemic and global conflicts. In terms of construct validity and potential translational relevance, the six week duration of isolation is equivalent to approximately 4.5% of the average rat lifespan which equates to approximately 3.5 years in humans, based on average life expectancies in Europe and the USA. Our paradigm prevents physical contact and play without abolishing visual and auditory contact, and we feel it is plausible that sectors of the population experience psychosocial stress of a similar nature and duration.

Because the response to isolation can be influenced by multiple factors (including rat strain, age at weaning, type of caging, availability of bedding, frequency of handling) and even vary between cohorts in the same laboratory (Heidbreder et al., 2000; Fone and Porkess, 2008) we were mindful of the need to verify syndrome development in the primary cohort prior to RNA-Seq. Regulatory considerations prevented examination of isolation-induced behavioural changes in these animals, but the increase in body weight that we observed has also been reported for numerous cohorts of isolates dating back over 50 years (e.g. Shelley, 1965; Jahng et al., 2012; Simpson et al., 2012; Gilabert-Juan et al., 2013; Kaneda et al., 2021; Tzeng et al., 2023). Crucially these increases mirror the effect of social adversity on body weight in humans (Hemmingsson et al., 2023) and have been attributed to reduced energy expenditure in the absence of peer interaction, as well as increased food and water intake during the light phase of the cycle which points to blunting of normal diurnal variation (Morgan and Einon, 1975; Fiala et al., 1977). It may also stem from increased incentive motivation, since isolates show different food preferences (Hall et al., 1997a; Sahakian et al., 1982) and response rates in sucrose reward tasks (Hall et al., 1997b). Isolation-induced increases in body weight have historically been accompanied by an array of behavioural changes, including locomotor hyperactivity in a novel arena (thought to reflect mesolimbic dopamine hyperactivity relevant to positive symptoms of schizophrenia), increased anxiety in an open field (Cao et al., 2021), cognitive deficits during novel object recognition testing, reduced sociability during social interaction testing, increased immobility during the forced swim test (suggestive of a depressive-like state) and increased plasma corticosterone that indicates perturbation of the hypothalamic-pituitary-adrenal axis (Jahng et al., 2012; Kaneda et al., 2021; King et al., unpublished observations). Indeed in the current validation cohort, where behavioural testing was feasible, the isolation-induced increase in body weight was once again accompanied by an anxiogenic phenotype and impaired novel object recognition. Furthermore, the subjective increases defensive behaviour and handling reactivity noted here have been reliably detected in our previous studies, where formal behavioural assessment (by observers unaware of housing allocation) again confirmed robust locomotor and cognitive changes (e.g. Jones et al., 2011; Dunphy-Doherty et al., 2018; Shortall et al., 2018; Shortall et al., 2020). Defence behaviours have also been reported by others and are accompanied by exacerbated endocrine (corticosterone) and autonomic (heart rate) indicators of the stress response (Toth et al., 2011). Changes to specific markers within the PFC are generally only reported in individual studies on the associated hypothesis, but our ability to selectively replicate reduced levels of both ErbB4 and NR2B mRNA (without influencing levels of GAD67 or parvalbumin mRNA) provides additional reassurance that isolation-induced changes in the current RNA-Seq cohort encompass those previously reported within our target brain region (Gilabert-Juan et al., 2013; Begni et al., 2020; Loureiro et al., 2020). These changes were also broadly in line with those in other models for schizophrenia and depression (e.g. involving gestational administration of an antimitotic agent, or adulthood exposure to chronic unpredictable mild stress or NMDA receptor antagonists; Gulchina et al., 2017; Shepard and Coutellier, 2018; Xie et al., 2020).

RNA-Seq revealed a higher number of down-regulated than up-regulated DEGs following isolation rearing. This is not a universal finding following all stressors (e.g. Olave et al., 2022), but is consistent with frontal cortical, hippocampal and amygdaloid responses to other social manipulations (e.g. Sun et al., 2018; Alugubelly et al., 2019; Alyamani et al., 2022; Green et al., 2021) as well as adrenal responses to isolation rearing (Jacobson et al., 2018). Over two thirds of DEGs and one third of enriched functional terms linked to prolonged social isolation of rats were also identified in post-mortem PFC samples from patients with schizophrenia, BD, MDD and/or ASD. Although post-weaning isolation rearing was originally regarded as a model for schizophrenia our findings echo the fact that social stress predisposes to a far broader array of psychiatric illnesses, and further occurs in many patients as a consequence of their symptoms. Current findings also fit with sharing of both symptoms (e.g. psychomotor change, cognitive impairment, social dysfunction) and genetic architecture between illnesses. For example, one study (involving over 260,000 patients from 25 diagnostic groups, versus 860,000 controls) revealed genetic correlations across psychiatric conditions, including of schizophrenia with ASD, BD, MDD, as well as of MDD with BD (Brainstorm Consortium, 2018). This is reinforced by recent transcriptomic profiling (of ∼2600 patients versus ∼2000 controls) that again found correlations between schizophrenia and ASD or BD, as well as between ASD and MDD (Sadeghi et al., 2022).

Interestingly, the 12 up-regulated genes shared between isolation rearing and the various disorders encode a diverse array of proteins involved in metabolism (*Mri1*), mitochondrial function (*Atp5l*, *Mrpl50*, *Ndufb6*), oxidative stress (*Sod1*), microtubule organisation (*Bloc1s2*, *Rita1*), ion transport (*Fxyd7*) and presynaptic vesicle trafficking and/or neurotransmitter release (*Hrh3*, *Snca*). The 116 shared down-regulated genes encode numerous phosphatases and kinases (e.g. *Dusp* family members, *Prkca*), transcription factors (*Egr* family members) and transcription repressors (*Mnt*, *Trps1*) as well as muscarinic (*Chrm4*) and NMDA (*Grin2b*) receptors, Ca^2+^ (*Cacna1e*) and K^+^ (*Kcnj6*) channels. They also encode multiple proteins crucial for neurodevelopment, including those involved in cortical neurogenesis (*Hipk2*), neuronal migration or maturation (*Dcx*, *Nav3*, *Nfib*, *Taok1*), synapse formation or plasticity (*Arhgef12*, *Itpr1*, *Nr4a1*) and turnover of glutamate receptors in response to synaptic activity (*Dlgap2*) (e.g. Rasmussen et al., 2017; Jeanneteau et al., 2018; van Woerden et al., 2021). The diversity of stress-induced changes is supported by GO enrichment analysis, which linked our DEGs to regulation of cellular, metabolic and biosynthetic processes that are fundamental to many cell types and neurotransmitter systems. Similarly, pathway analysis revealed predicted inhibition of numerous signalling cascades likely to underlie behavioural, structural and neurochemical responses to social isolation, including impaired LTP (Quan et al., 2010). We also identify predicted inhibition of additional pathways that are known to be dysregulated following other forms of stress and may represent therapeutic targets in psychiatric disorders but have not yet been associated with brain regional responses to social isolation, including aryl hydrocarbon receptor signalling (Li et al., 2022). The current down-regulation of *Ep300* and *Ncoa3* transcription regulator genes is predicted to inhibit activity of the aryl hydrocarbon receptor, a ligand-activated transcription factor stimulated by numerous exogenous and endogenous molecules including gut microbiome metabolites (Wei et al., 2021). Aryl hydrocarbon receptor inhibition reduces expression of genes encoding xenobiotic metabolising enzymes like *Cyp1b1*, and has the potential for widespread downstream consequences encompassing apoptosis and oestrogen metabolism (for review see Ojo and Tischkau, 2021). We acknowledge that the shared DEGs discussed above only represent a small proportion (<5%) of the total number in disease states. However, it is important to remember that psychiatric and neurodevelopmental illnesses are complex disorders with multiple genetic and environmental triggers, and it is therefore interesting to note the number, identity and functional roles of shared DEGs that can actually be induced by social isolation as the sole stressor. In this context we feel that current data support use of post-weaning isolation to investigate disease neurobiology, and indeed controlled laboratory studies like this are vital to tease out which aspects of pathology might be due to which risk factors, and ultimately enable a more personalised approach to medicine (Dhindsa et al., 2021).

Some consequences of post-weaning isolation rearing (or social isolation of adult rats) can be reversed by environmental enrichment or exercise (e.g. Hellemans et al., 2004; Ishikawa and Ishikawa, 2013; Park et al., 2020; Guven et al., 2022), and non-pharmacological approaches can also benefit some patient populations (e.g. Solmi et al., 2023; Tseng et al., 2023). In terms of pharmacological treatment, we report a number of potentially druggable targets although some (e.g. *Hipk2* and *Nfib*) can be quickly ruled out on the basis of oncogenic potential (Yang et al., 2021; Conte et al., 2023). Amongst the remainder we highlight nine targets with small molecule ligands or antibody-based therapeutics that would be predicted to oppose isolation-induced (and thus also disease-induced) alterations. There are antagonists or inhibitors for protein products of *Hrh3*, *Snca* and *Sod1* genes and agonists or activators for products of *Chrm4*, *Klf2*, *Lrrk2*, the *Nr4a* gene family and *Prkca*. Six of these nine targets (67%) were confirmed by qRT-PCR; *Hrh3*, *Sod1*, *Chrm4*, *Lrrk2*, *Nr4a1* and *Prkca*, while a further two (22%) were close to significance; *Klf2* and *Nr4a3*. This level of replication is particularly reassuring since we were not simply comparing RNA-Seq and qRT-PCR analyses of the same tissue samples (which is generally accepted to produce 80–85% agreement; Everaert et al., 2017), but instead performing completely independent verification using separate cohorts of animals housed and examined at different institutions by different researchers. Some of the highlighted pharmacological agents have already undergone pre-clinical and clinical evaluation for relevant disorders. For instance, selective H_3_ receptor antagonists reversed sensorimotor gating deficits in isolation-reared rats (Southam et al., 2009) and had small-scale positive effects on the CogState Schizophrenia Battery composite score during a double-blind placebo-controlled trial (Jarskog et al., 2015), while a dual H_1_ receptor agonist and H_3_ antagonist improved MATRICS Consensus Cognitive Battery composite scores (Wang et al., 2021). With the exception of a recent case report describing improvement of both negative and cognitive symptoms (Naguy et al., 2021) other clinical findings in schizophrenia were negative (e.g. Egan et al., 2013; Haig et al., 2014) but there has been recent interest in revisiting pre-clinical observations in stress-related depression (for review see Alhusaini et al., 2022), where clinical evaluations do not appear to have taken place. Separate to this the M_1_ and M_4_ receptor agonist xanomeline improved Brief Psychiatry Rating Scale and Positive and Negative Syndrome Scale total scores (Shekhar et al., 2008) but development for schizophrenia was limited by peripheral adverse effects. Bristol Myers Squibb and Karuna Therapeutics have ignited renewed interest in xanomeline through their well-tolerated formulation with the peripherally-restricted muscarinic receptor antagonist, trospium, known as KarXT. Phase 2 and 3 clinical trials demonstrated antipsychotic activity in schizophrenia patients together with effects on negative symptoms (Correll et al., 2022; Paul et al., 2022; Sauder et al., 2022; Horan et al., 2024; Kaul et al., 2024), resulting in KarXT receiving very recent Food and Drug Administration (FDA) approval (Kingswell, 2024). In addition, more selective M_4_ agonists or positive allosteric modulators developed by Pfizer (Yang et al., 2019; Butler et al., 2024) and Sosei Heptares (e.g. NBI-1117568; Sosei Group Corporation 2022) may provide similar efficacy with further reduced adverse events. Preclinical studies show the atypical antidepressant agomelatine (which is a melatonin MT1 and MT2 receptor agonist and 5-HT_2C_ receptor antagonist) can reverse chronic mild stress-induced increases in *Sod1* expression (Wigner et al., 2020) and more selective downregulation might be possible via antisense oligonucleotides (Miller et al., 2022) that were safely administered in phase 2–3 trials for neurodegenerative disorders (e.g. Tabrizi et al., 2019) and subsequently approved by the FDA and European Medicines Agency (EMA; Cantara et al., 2024). Several remaining approaches suggested by our RNA-Seq data are yet to be investigated for psychiatric or neurodevelopmental disorders. Key priorities should perhaps be nuclear receptor 4A1 (NR4A1) activators (Bridi et al., 2017) given that *Nr4a1* down-regulation in isolates was greatest in terms of fold-change, and it was included in the 13% of DEGs shared with three of the four selected illnesses (namely schizophrenia, MDD and BP). Furthermore, *Nr4a1* downregulation has been shown to trigger microglial activation and increased synaptic pruning (Rothe et al., 2017; Han et al., 2022), whereas the NR4A1 receptor agonist cytosporone B reduces neuroinflammation in models of neurodegenerative illness (Yu et al., 2022). It is also noteworthy that infusions of the rapid acting antidepressant ketamine upregulate *Nr4a1* expression across multiple brain regions in normal rats (Kim et al., 2022) and the antioxidant N-acetylcysteine has similar effects in rats exposed to acute restraint stress, leading to *Nr4a1* modulation being proposed as a mechanism for promoting resilience (Brivio et al., 2023). PKC activators are also a high priority given the involvement of *Prkca* in 13 of the 18 signalling pathways disrupted by isolation rearing and identification of PKC inhibition as a potential driver for multiple isolation-induced transcriptional changes. PKC activators were originally considered for Alzheimer's disease (Talman et al., 2016) and nanobodies that act as allosteric activators of leucine-rich repeat kinase 2 (LRRK2) have been discussed in the context of Parkinson's disease (Singh et al., 2022). Isolation-induced down-regulation of *Klf2* just failed to reach statistical significance during our qRT-PCR validation of RNA-Seq data, but Krüppel-like factor 2 (KLF2) activators have anti-inflammatory effects in vascular cells (Xu et al., 2017; Lei et al., 2022) and any demonstration of similar properties in brain tissue would still be of potential interest.

In summary, the current RNA-Seq analysis of transcriptomic changes in the PFC of isolation-reared rats revealed a total of 128 DEGs that mirrored those in PFC tissue from patients with psychiatric illnesses that can be triggered by psychosocial stress and/or neurodevelopmental disorders featuring social deficits. We acknowledge that the current analysis was restricted to male rats, and this decision was originally taken because isolation-rearing has traditionally been viewed as a rodent model for schizophrenia (for reviews see Fone and Porkess, 2008; Jones et al., 2011). That particular illness has very well-recognised biological sex differences, with males exhibiting an earlier age at symptom onset, worse spectrum of symptoms and poorer response to existing medications. Recent transcriptomic analyses of the rodent prefrontal cortex in response to other forms of stress have revealed interesting sexual dimorphisms (Zhang et al., 2023) that warrant further exploration in the context of social isolation and may offer insight into gender differences in susceptibility versus resilience to stress and mental illness. We also acknowledge that the bulk PFC tissue analysed here comprises multiple sub-regions each containing different subtypes of excitatory and inhibitory neurons as well as astrocytes, oligodendrocytes, microglia and vascular cells. It is therefore possible that altered gene expression in one cell type could be masked by an opposing change in others (Darmanis et al., 2015; McKenzie et al., 2018), and future application of single-cell RNA-Seq (Cai et al., 2022) could reveal these differential effects to shed even more light on neurobiological responses to social isolation. For example, single-cell analysis of human RNA-Seq datasets has recently revealed differential findings in astrocytes versus excitatory neurones versus inhibitory neurones in PTSD (Chatzinakos et al., 2023), and shown cell type-specific clustering of different risk genes in excitatory neurones versus inhibitory neurones versus oligodendrocyte precursors in schizophrenia (Bryois et al., 2022). Our findings could also have been extended by examination of epigenetic changes regulating gene expression (Gräff et al., 2011) as well as protein expression of DEGs (including those in the aryl hydrocarbon receptor signalling pathway), given that gene and protein expression might not always correlate (Kosti et al., 2016). Nevertheless, present findings extend our confidence in the face and construct validity of the isolation rearing paradigm for studying the neurobiology of stress-related mental illnesses. Furthermore we identify pharmacological targets like the M_4_ receptor that have already been investigated, plus others like NR4A1 receptors that may merit new attention. Crucially pre-clinical evaluation of compounds intended for management of psychosocial stress-related mental illnesses should be conducted in relevant stress-induced models, and any subsequent clinical trials should involve patient stratification based on life history.

## CRediT authorship contribution statement

**Jen-Yin Goh:** Writing – original draft, Investigation, Formal analysis, Data curation. **Patricia Rueda:** Formal analysis, Data curation. **Joy Taylor:** Investigation, Data curation, Formal analysis. **Alex Rathbone:** Investigation, Data curation, Formal analysis, Writing – original draft. **Daniel Scott:** Writing – review & editing, Formal analysis, Data curation. **Christopher J. Langmead:** Writing – review & editing, Supervision, Funding acquisition, Conceptualization. **Kevin C.F. Fone:** Writing – review & editing, Supervision, Funding acquisition, Conceptualization. **Gregory D. Stewart:** Writing – review & editing, Supervision, Conceptualization. **Madeleine V. King:** Writing – review & editing, Writing – original draft, Supervision, Funding acquisition, Formal analysis, Data curation, Conceptualization.

## Declaration of competing interest

The authors declare that they have no known competing financial interests or personal relationships that could have appeared to influence the work reported in this paper.

## Data Availability

Data will be made available on request.
